# The impact of improved detection and treatment of isoniazid resistant tuberculosis on prevalence of multi-drug resistant tuberculosis: A modelling study

**DOI:** 10.1371/journal.pone.0211355

**Published:** 2019-01-24

**Authors:** Kamila Romanowski, Jonathon R. Campbell, Olivia Oxlade, Federica Fregonese, Dick Menzies, James C. Johnston

**Affiliations:** 1 TB Services, BC Centre for Disease Control, Vancouver, British Columbia, Canada; 2 McGill International TB Centre, Montreal, Quebec, Canada; 3 Division of Respiratory Medicine, Department of Medicine, McGill University, Quebec, Canada; 4 Division of Respiratory Medicine, Department of Medicine, University of British Columbia, Canada; National Institute of Allergy and Infectious Diseases, UNITED STATES

## Abstract

**Introduction:**

Isoniazid-resistant, rifampin susceptible tuberculosis (INHR-TB) is the most common form of drug resistant TB globally. Treatment of INHR-TB with standard first-line therapy is associated with high rates of multidrug resistant TB (MDR-TB). We modelled the potential impact of INHR-TB detection and appropriate treatment on MDR-TB prevalence.

**Methods:**

A decision analysis model was developed to compare three different strategies for the detection of TB (AFB smear, Xpert MTB/RIF, and Line-Probe Assays (LPA)), combined with appropriate treatment. The population evaluated were patients with a globally representative prevalence of newly diagnosed, drug-susceptible (88.6%), isoniazid-resistant (7.3%), and multidrug resistant (4.1%) pulmonary TB. Our primary outcome was the proportion of patients with MDR-TB after initial attempt at diagnosis and treatment within a 2-year period. Secondary outcomes were the proportion of i) individuals with detected TB who acquired MDR-TB ii) individuals who died after initial attempt at diagnosis and treatment.

**Results:**

After initial attempt at diagnosis and treatment, LPA combined with appropriate INHR-TB therapy resulted in a lower proportion of prevalent MDR-TB (1.61%; 95% Uncertainty Range (UR: 2.5^th^ and 97.5^th^ percentiles generated from 10 000 Monte Carlo simulation trials) 1.61–1.65), when compared to Xpert (1.84%; 95% UR 1.82–1.85) and AFB smear (3.21%; 95% UR 3.19–3.26). LPA also resulted in fewer cases of acquired MDR-TB in those with detected TB (0.35%; 95% UR 0.34–0.35), when compared to Xpert (0.67%; 95% UR 0.65–0.67) and AFB smear (0.68%; 95% UR 0.67–0.69). The majority of acquired MDR-TB arose from the treatment of INHR-TB in all strategies. Xpert-based strategies resulted in a lower proportion of death (2.89%; 95% UR 2.87–2.90) compared to LPA (2.93%; 95% UR 2.91–2.94) and AFB smear (3.21%; 95% UR 3.19–3.23).

**Conclusion:**

Accurate diagnosis and tailored treatment of INHR-TB with LPA led to an almost 50% relative decrease in acquired MDR-TB when compared with an Xpert MTB/RIF strategy. Continued reliance on diagnostic and treatment protocols that ignore INHR-TB will likely result in further generation of MDR-TB.

## Introduction

Drug-resistant tuberculosis (TB) is a significant threat to TB elimination.[1] While research and public health action in this area has predominately focused on multi-drug resistant TB (MDR-TB; defined as TB resistant to at least both rifampicin and isoniazid) and extensively drug-resistant TB (XDR-TB; defined as MDR-TB with additional resistance to a fluoroquinolone and one or more second-line injectables), isoniazid-resistant, rifampin susceptible TB (INHR-TB) accounts for more than one third of all drug-resistant isolates.[2,3] Between 1994 and 2009, some form of isoniazid resistance was detected in 44.9% of incident TB cases in eastern Europe and in 14% of incident cases all other regions combined.[4] According to World Health Organization (WHO) estimates, in 2017 the global average of INHR-TB was detected in 7.3% of new and 14.0% of previously treated TB cases, respectively.^1^

A recent systematic review and meta-analysis highlighted the high rate of adverse treatment outcomes when INHR-TB was undetected and treated with standardised first-line regimens.[5] Treatment of INHR-TB with WHO standard first-line TB therapy for new cases resulted in high proportions of failure (11%), relapse (10%), and acquired rifampin resistance (8%).[5] Alternative regimens, with extended durations of pyrazinamide and/or rifampin, were associated with significantly lower proportions of adverse outcomes. Most notably, the proportion of acquired rifampin resistance was <1% when INHR-TB was treated with 6–9 months of rifampin, pyrazinamide, and ethambutol (6-9REZ).[5]

Most laboratories in low- and middle-income countries have limited capacity to diagnose INHR-TB.[1] Drug susceptibility testing (DST) was performed on less than 10% of the 10.4 million people with TB in 2015,[6] and since the most commonly used rapid diagnostic test—the nucleic acid amplification based Xpert MTB/RIF system—detects only rifampin resistant TB (RIFR-TB) as a proxy for MDR-TB, INHR-TB will continue to be missed.[7,8] Alternative rapid diagnostic tests, endorsed by WHO since 2008, are line probe assays (LPA), which can detect both INHR-TB and RIFR-TB.[9] Detection of INHR-TB, combined with directed INHR-TB therapy, has the potential to improve outcomes in people with INHR-TB and may reduce the generation of new MDR-TB strains, but the impact of enhanced INHR-TB detection and treatment on MDR-TB prevalence at a population level is unknown.

In this study, we aimed to estimate the impact of introducing INHR-TB detection and directed therapy on a population with a globally representative distribution of drug resistant TB by comparing outcomes from three different diagnosis and treatment strategies. Our primary objective was to estimate the proportion of prevalent MDR-TB in the total population within a 2-year period. Our secondary objectives were to estimate i) the proportion of individuals with detected TB who acquired MDR-TB ii) the proportion of individuals who died after initial attempt at diagnosis and treatment.

## Methods

### Population and setting

We simulated a hypothetical cohort of HIV negative patients with a globally representative prevalence of newly diagnosed, drug-susceptible, isoniazid resistant, and multi-drug resistant (88.6%, 7.3%, and 4.1% respectively) smear positive and negative pulmonary TB as the study population. Global resistance prevalence was based on 2017 WHO estimates.[1]

### Decision analysis model

**Fig 1:**
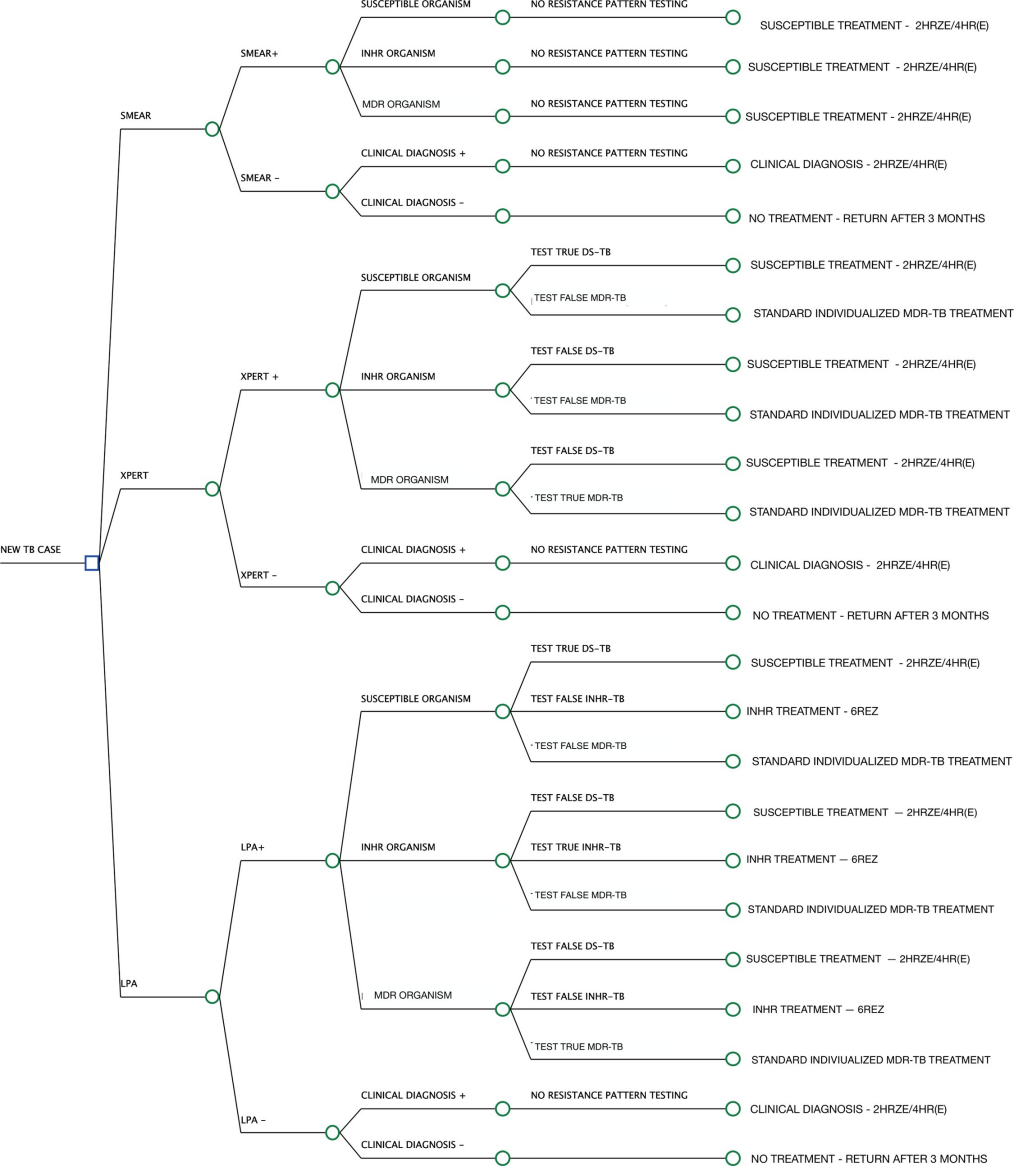
Simplified schematic of model. This diagram depicts a simplified representation of the decision analysis model. The square indicates a decision node and the circles indicate probability nodes. Three different diagnostic and treatment strategies were considered over a two-year period in the decision analysis model: (1) AFB smear with WHO standard first line therapy for all people diagnosed with TB (2 months isoniazid, rifampin, pyrazinamide, ethambutol, followed by 4 months isoniazid, rifampin (2HRZE/4HR)); (2) Xpert MTB/RIF with standardized WHO recommended MDR-TB therapy for those with detected MDR-TB, and 2HRZE/4HR for the remainder; (3) LPA with a daily regimen of 6 months of rifampin, ethambutol, and pyrazinamide with or without isoniazid (6(H)REZ) for detected INHR-TB, standardized WHO recommended MDR-TB therapy for those with detected MDR-TB, and 2HRZE/4HR for the remainder. TB (tuberculosis); INHR (isoniazid resistant); MDR (multi-drug resistant); LPA (Line Probe Assay).

To evaluate impact of improved detection and treatment of isoniazid resistant tuberculosis on prevalence of multi-drug resistant tuberculosis we constructed a decision analysis model using TreeAge Pro software (Version 2017, TreeAge Software Inc., Williamstown, MA), following steps outlined by *Bae et al*.[10] We considered three different diagnostic and treatment strategies over a two-year period in our decision tree (Fig 1): (1) AFB smear with WHO standard first line therapy for all people diagnosed with TB (2 months isoniazid, rifampin, pyrazinamide, ethambutol, followed by 4 months isoniazid, rifampin (2HRZE/4HR)), (2) Xpert MTB/RIF with standardized WHO recommended MDR-TB therapy[11] for those with detected MDR-TB, and 2HRZE/4HR for the remainder, and (3) LPA with a daily regimen of 6 months of rifampin, ethambutol, and pyrazinamide with or without isoniazid (6(H)REZ) for detected INHR-TB, standardized WHO recommended MDR-TB therapy for those with detected MDR-TB, and 2HRZE/4HR for the remainder. For each step of the tree, we input corresponding probabilities or endpoint utility values. Data on the diagnostic accuracy of tests and likelihood of treatment outcomes was obtained from systematic reviews and published data (Table 1)[12–14] Once all values were assigned, we calculated the likelihood of each outcome occurring under deterministic parameter conditions.

**Table 1:** Summary of model inputs.

Description	Parameter Estimate	PSA		Source
		Distribution	95% CI	
**Population proportions**				
Proportion of patients with INHR-TB	0·07	Beta	(0·06, 0·09)	^1^
Proportion of patients with RIFR-TB	0.04	Beta	(0.03, 0.05)	^1^
**Diagnostic parameters**				
**Sensitivity for diagnosing pulmonary TB**				
AFB Smear microscopy (3 slides)	0.70	Beta	(0.62, 0.78)	^12^
Xpert MTB/RIF	0.89	Beta	(0.86, 0.92)	^13^
LPA	0.85	Beta	(0.73, 0.94)	^14^
**Sensitivity for detecting rifampin resistance**				
Xpert MTB RIF	0.95	Beta	(0.91, 0.98)	^13^
LPA	0.97	Beta	(0.96, 0.98)	^14^
**Specificity for detecting rifampin resistance**				
Xpert MTB RIF	0.98	Beta	(0.97, 0.99)	^13^
LPA	0.99	Beta	(0.98, 0.99)	^14^
**Sensitivity for detecting isoniazid resistance**				
LPA	0.90	Beta	(0.89, 0.92)	^14^
**Specificity for detecting isoniazid resistance**				
LPA	0.99	Beta	(0.99, 0.99)	^14^
**Clinical diagnosis, for those with a false negative diagnostic test**				
AFB Smear	0.30	Beta	(0.21, 0.39)	^19^
Xpert MTB/RIF	0.05	Beta	(0.01, 0.11)	^19^
LPA	0.05	Beta	(0.01, 0.11)	Model assumption
**Treatment outcomes for detected or clinically diagnosed TB**				
**Susceptible organism treated with standard initial treatment [2HRZE/4HR(E)]**				
Death	0.02	Beta	(0.02, 0.03)	^17^
Treatment failure	0.03	Beta	(0.02, 0.03)	^5^
Relapse	0.06	Beta	(0.05, 0.06)	^5^
Acquired any drug resistance due to treatment failure	0.13	Beta	(0.08, 0.19)	^5^
Acquired any drug resistance due to relapse	0.02	Beta	(0.00, 0.04)	^5^
Proportion of any acquired drug resistance that is multidrug resistant	0.46	Beta	(0.38, 0.54)	^5^
**Susceptible organism treated with INHR-TB treatment [6(H)REZ]**				
Death	0.03	Beta	(0.02, 0.03)	Model assumption
Treatment failure	0.01	Beta	(0.01, 0.02)	^5^
Relapse	0.06	Beta	(0.04, 0.07)	^5^
Acquired any drug resistance due treatment failure	0.01	Beta	(0.00, 0.04)	^5^
Acquired any drug resistance due to relapse	<0.00	Beta	(<0.00, <0.00)	^5^
Proportion of any acquired drug resistance that is multidrug resistant	0.27	Beta	(0.09, 0.51)	^5^
**INHR organism treated with standard initial treatment [2HRZE/4HR(E)]**				
Death	0.03	Beta	(0.02, 0.03)	Model assumption
Treatment failure	0.11	Beta	(0.08, 0.15)	^16^
Relapse	0.14	Beta	(0.10, 0.19)	^16^
Acquired multidrug resistance due to treatment failure	0.53	Beta	(0.33, 0.74)	^16^
Acquired multidrug resistance due to relapse	0.09	Beta	(0.01, 0.26)	^16^
**INHR organism treated with INHR-TB treatment [6(H)REZ]**				
Death	0.03	Beta	(0.02, 0.03)	^16^
Fail	0.04	Beta	(0.04, 0.05)	^16^
Relapse	< 0.00	Beta	(0.00, 0.01)	^16^
Acquired multidrug resistance due to treatment failure	0.40	Beta	(0.39, 0.56)	^16^
Acquired multidrug resistance due to relapse	0.20	Beta	(0.01, 0.53)	^16^
**MDR organism treated with standard initial treatment [2HRZE/ 4HR(E)]**^**a**^				
Death	0.14	Beta	(0.03, 0.26)	^18^
Treatment failure	0.26	Beta	(0.21, 0.31)	^18^
Relapse	0.26	Beta	(0.21, 0.31)	Model assumption
**MDR organism treated with WHO standard individualized MDR-TB treatment**^**b**^				
Death	0.08	Beta	(0.05, 0.11)	^18^
Treatment failure	0.05	Beta	(0.03, 0.07)	^18^
Relapse	0.02	Beta	(0.01, 0.03)	^26^
**Outcomes for untreated TB**				
Death^c^	0.05	Beta	(0.03, 0.06)	^21^

^a ^Assumed identical outcomes for MDR organism treated with INHR-TB therapy

^b^ Assumed identical outcomes for susceptible organism/INHR organism treated with MDR-TB treatment

^c^ Over a 2 year post-treatment period, assuming smear negative disease

The initial prevalence of DS-TB, INHR-TB, and MDR-TB in the simulated population was obtained from global estimates reported in the 2017 WHO TB report.[15] INHR-TB strains were defined as isoniazid resistant and rifampin-susceptible, with or without resistance to other first line drugs as described by Gegia *et al*.[5] MDR-TB strains were defined as either resistance to rifampin and isoniazid, or rifampin resistance with or without additional resistance, as defined by the WHO[1]. Secondary cases due to transmission were not considered in this model.

The simulated patient pathway began with an initial TB detection attempt using one of the three diagnostic strategies. All individuals with a positive diagnostic test received treatment, based on their diagnostic test result (Table 2). Treatment was initiated based on diagnostic results, regardless of the true underlying resistance pattern (e.g. false positive MDR-TB results were treated as MDR-TB, and false negative INHR-TB results were treated as DS-TB, etc.). Treatment outcomes were acquired from published studies and are detailed in Table 2.[5,16–18] Five-treatment outcomes were possible for those who initiated treatment: treatment success, treatment failure, relapse within two years post treatment completion, death during treatment, or default. Default represented a combined programmatic outcome of default, loss to follow-up, or transferred out. Patients with drug susceptible or INHR-TB experiencing failure or relapse were at risk of acquiring MDR-TB if treated with a rifampin-containing regimen. Once acquired, drug resistance could not resolve spontaneously.

**Table 2:** Diagnostic and treatment strategies.

Strategy	RIFR-TB Detected	INHR-TB Detected	Treatment Regimen
**AFB Smear**	N/A	N/A	2HRZE/ 4HR(E)^a^
**Xpert MTB/RIF**	No	N/A	2HRZE/ 4HR(E)^a^
	Yes	N/A	MDR-TB treatment^b^
**Line Probe Assay**	No	No	2HRZE/ 4HR(E) ^a^
	No	Yes	6(H)REZ^c^
	Yes	Yes	MDR-TB treatment^b^
	Yes	No	MDR-TB treatment^b^

^a^ 2HRZE/4HR: 2 months of isoniazid, rifampin, pyrazinamide, +/-ethambutol followed by 4 months of isoniazid, rifampin, +/-ethambutol

^b^WHO standard individualized MDR-TB therapy

^c^6 months of rifampin, ethambutol, pyrazinamide, +/- isoniazid

Those with a false negative diagnostic test could receive a clinical diagnosis of TB and be given WHO standard first-line therapy. Based on WHO algorithms for systematic screening for active TB, we assumed of that 30% of AFB smear negative cases and 5% of Xpert MTB/RIF negative cases received a clinical diagnosis.[19] Due to limited data on clinical diagnosis with LPA, we also assumed that 5% of LPA cases received empiric treatment. TB cases that remained undiagnosed returned after three months for repeat testing, with 10% of undiagnosed cases becoming test-positive within that time frame.[20]

Those with a false negative diagnostic and no clinical diagnosis were simulated to a 2-year outcome of death due to TB or continued survival with no spontaneous regression or cure. We assumed a 4.9% risk of death over a 2-year period based on findings from *Tiemersma et al*., which estimate that 5-year mortality for smear-negative TB ranges from 8–15% (with a median of 12%).[21]

### Projected outcomes

Our primary outcome was the proportion of individuals in the cohort with prevalent MDR-TB after initial attempt at diagnosis and treatment within a 2-year time frame. Secondary outcomes were i) the proportion of individuals with detected TB who acquired MDR-TB ii) the proportion of individuals who died after initial attempt at diagnosis and treatment.

### Probabilistic sensitivity analysis

Probabilistic sensitivity analysis (PSA) was used to determine the extent of uncertainty from all model parameters combined. We performed 10 000 Monte Carlo trials to obtain 95% Uncertainty Ranges (UR; 2.5^th^ and 97.5^th^ percentiles) around point estimates for projected outcomes. As all model input parameters were proportions, parameters were fit to beta distributions for sampling during each of the PSA trials, with a mean corresponding to the deterministic parameter estimates. The 95% confidence intervals for each beta distribution were defined based on reported 95% confidence intervals or incidence of events from the literature, however when these were unavailable, expert opinion was used to estimate these values (S1 Table). PSA was also used to determine the proportion of simulations where a diagnostic scenario resulted in the fewest overall MDR-TB cases, fewest overall acquired MDR-TB cases, and fewest overall deaths.

### Threshold analyses

By fixing the input parameter of interest, we were able to completed multiple threshold analyses to determine the generalizability of our results to a wide variety of epidemiologic settings and resource levels. The first analysis evaluated a range of INHR-TB prevalence levels to estimate the likelihood a diagnostic strategy resulted in the fewest overall MDR-TB cases, fewest acquired MDR-TB cases, and fewest overall deaths at each level. The second analysis varied the prevalence of INHR-TB and MDR-TB to mimic various epidemiologic settings and evaluate which diagnostic strategy would result in the lowest prevalence of MDR-TB. The third analysis varied the proportion of patients receiving empiric treatment after a false negative result to evaluate if this impacted the conclusions from our primary analysis.

### Sensitivity analysis

For low-resource settings where an LPA-based strategy may not be possible to implement, we completed a sensitivity analysis to evaluate the utility of an Xpert-based assay that could also detect INH resistance using model inputs from the recent findings of Xie *et al*.[8].

## Results

After initial attempt at diagnosis and treatment, the proportion of individuals in the cohort with MDR-TB after a 2-year period was 1.61% (95% UR 1.61–1.65) using an LPA-based strategy, compared to 1.84% (95% UR 1.82–1.85) in the Xpert MTB/RIF-based strategy, and 3.21% (95% UR 3.19–3.26) in the smear-based strategy (Table 3). LPA-based diagnosis and treatment strategies also resulted in fewer cases of acquired MDR-TB, with 0.35% (95% UR 0.34–0.35) of those with detected TB acquiring MDR-TB compared to 0.67% (95% UR 0.65–0.67) in the Xpert MTB/RIF and 0.68% (95% UR 0.67–0.69) in the AFB smear strategies (Table 4). Xpert-based strategies resulted in a slightly lower proportion of overall deaths (2.89%; 95% UR 2.87–2.90) compared to LPA (2.93%; 95% UR 2.91–2.94) and AFB smear strategies (3.21%; 95% UR 3.19–3.23)(Table 5).

**Table 3:** Prevalence of MDR-TB in population after initial attempt at diagnosis and treatment, per 100 000 individuals with TB.

	AFB Smear	Xpert MTB/RIF	LPA
**Total MDR-TB**	3212	1835	1614
% of total population	3.21	1.84	1.61
**Breakdown of total MDR-TB**			
Ongoing undetected MDR-TB	737	367	500
MDR-TB cases who failed or relapsed	1190	292	265
MDR-TB cases who defaulted^a^	733	572	547
Acquired MDR-TB	552	604	302

^a^ Default represents a combined programmatic outcome of default, lost to follow-up or transferred out

**Table 4:** Total acquired MDR-TB in those with detected TB after initial attempt at diagnosis and treatment, per 100 000 individuals with TB.

	AFB Smear	Xpert MTB/RIF	LPA
Total TB detected or clinically diagnosed	81100	90594	87169
Total acquired MDR-TB	552	604	302
% of total TB detected	0.68	0.67	0.35
**Breakdown of total acquired MDR-TB resistance due to:** *(% of total acquired MDR-TB)*			
Susceptible organism treated with susceptible TB therapy	129	141	136
Susceptible organism treated with INHR-TB therapy	NA	NA	0
INHR organism treated with susceptible TB therapy	423	463	43
INHR organism treated with INHR-TB therapy	NA	NA	123

**Table 5:** Total death in population after initial attempt at diagnosis and treatment, per 100 000 individuals with TB.

	AFB Smear	Xpert MTB/RIF	LPA
Total death	3206	2889	2924
% of total population	3.21	2.89	2.92
**Breakdown of total death**			
Death due to undetected TB	925	461	628
Death during TB therapy	2281	2428	2296

The majority of acquired MDR-TB arose from the treatment of INHR-TB in all three strategies (Table 4). Notably, treating undetected INHR organisms with 2HRZE/4HR generated approximately 80.0% of acquired MDR-TB in AFB Smear (76.67%; 95% UR 76.03–76.82) and Xpert MTB/RIF (76.59%; 95% UR 75.97–76.74) strategies. Using the LPA based strategy, 14.17% (95% UR 13.89–14.46) of acquired MDR was generated from treatment of INHR-TB with 2HRZE/4HR due to LPA false negatives. Treatment with 6(H)REZ was responsible for 40.98% (95% UR 40.56–41.41) of acquired MDR-TB in the LPA strategy.

### Probabilistic sensitivity analysis

PSA supported the results of our deterministic analysis (S2 Table). LPA-based diagnosis and treatment resulted in the fewest cases of acquired MDR-TB in those with detected TB and overall MDR-TB in 99.1% and 84.8% of probabilistic replications, respectively. Xpert-based diagnosis resulted in the fewest overall deaths in 55.8% of replications compared an LPA-based diagnosis in 43.5% of replications.

### Threshold analyses

**Fig 2:**
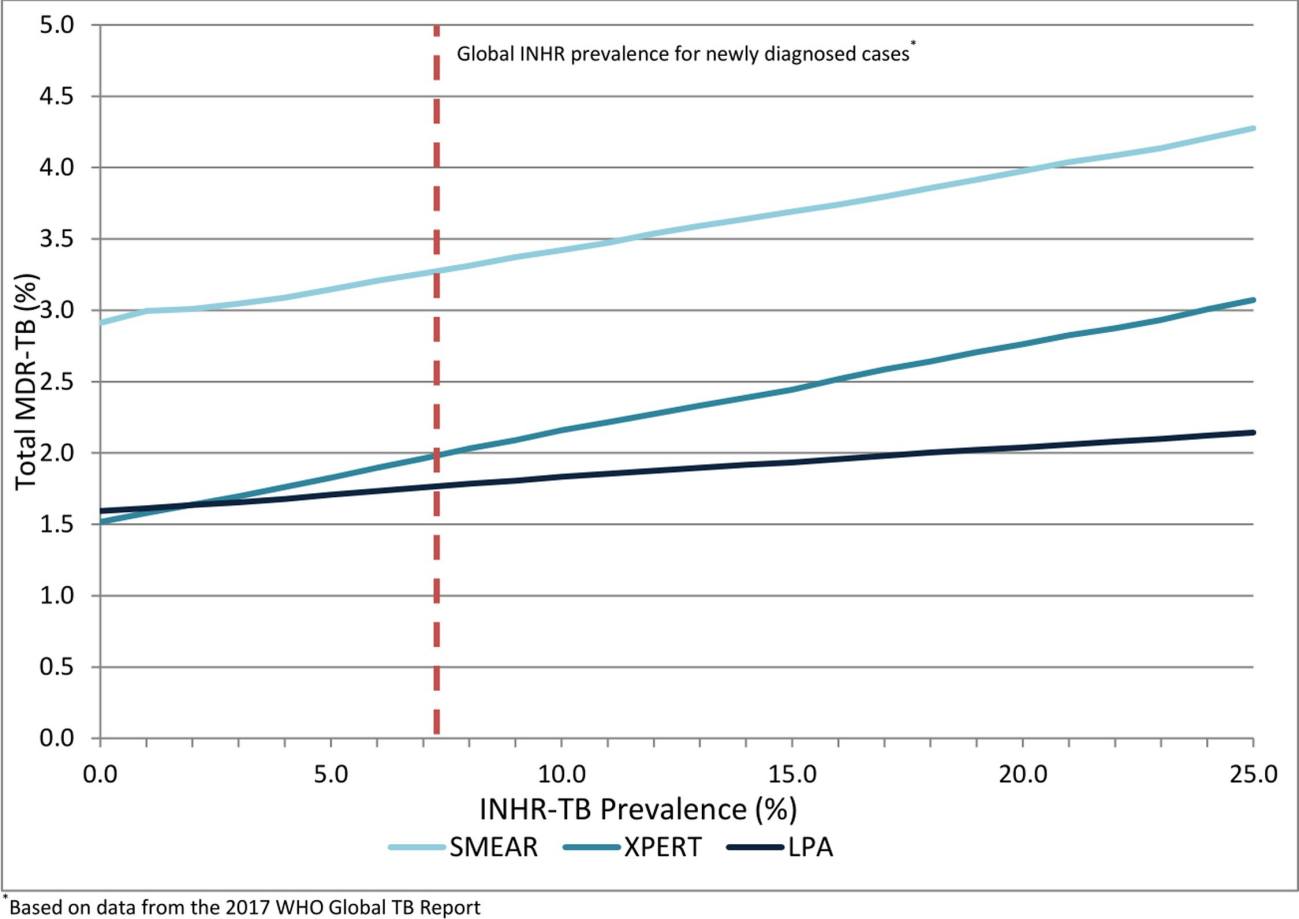
Total proportion of patients with MDR-TB after initial attempt at diagnosis and treatment, based on varying population INHR-TB prevalence.

**Fig 3:**
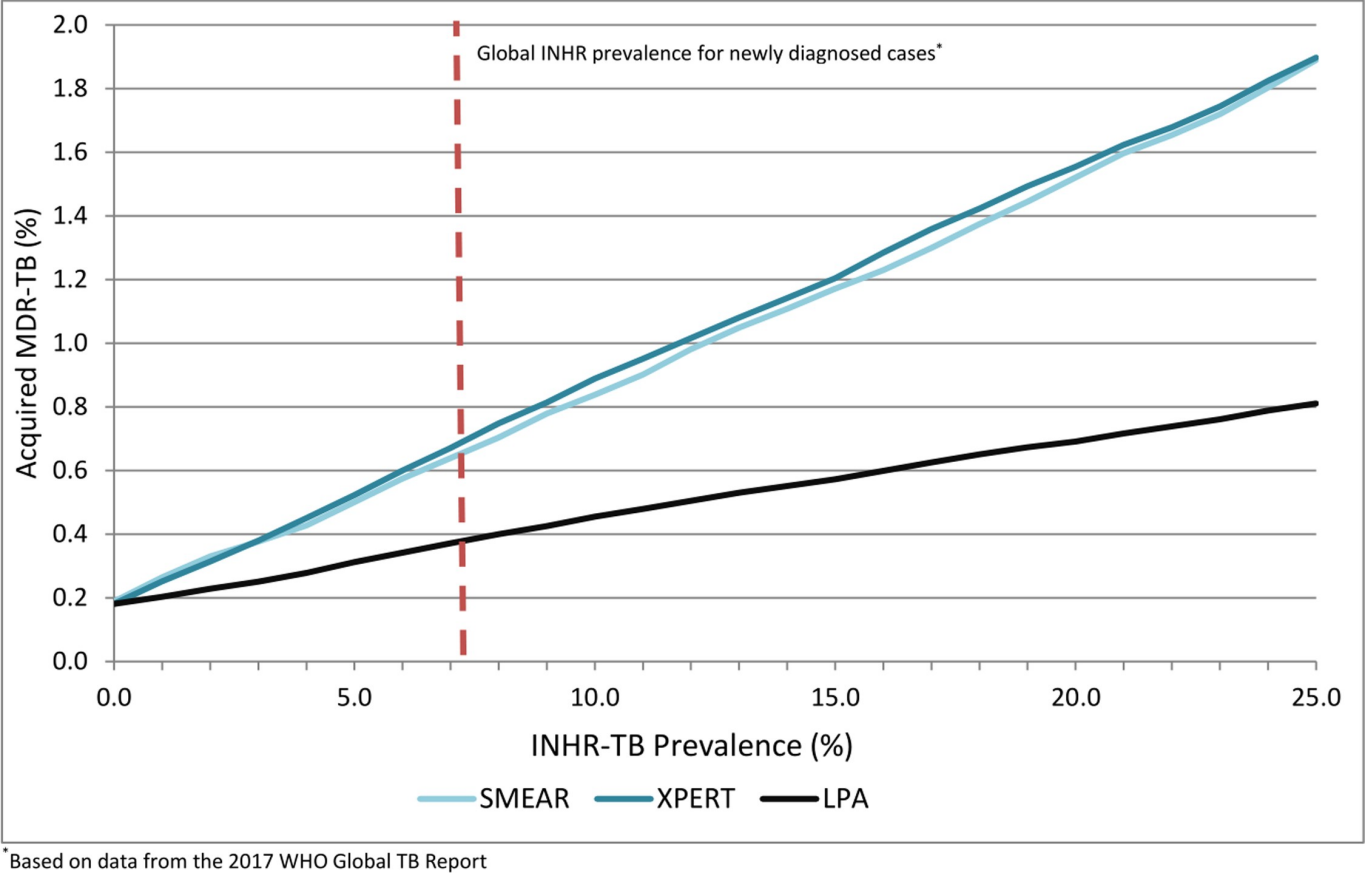
Proportion of patients with acquired MDR-TB after initial attempt at diagnosis and treatment, based on varying population INHR-TB prevalence.

**Fig 4:**
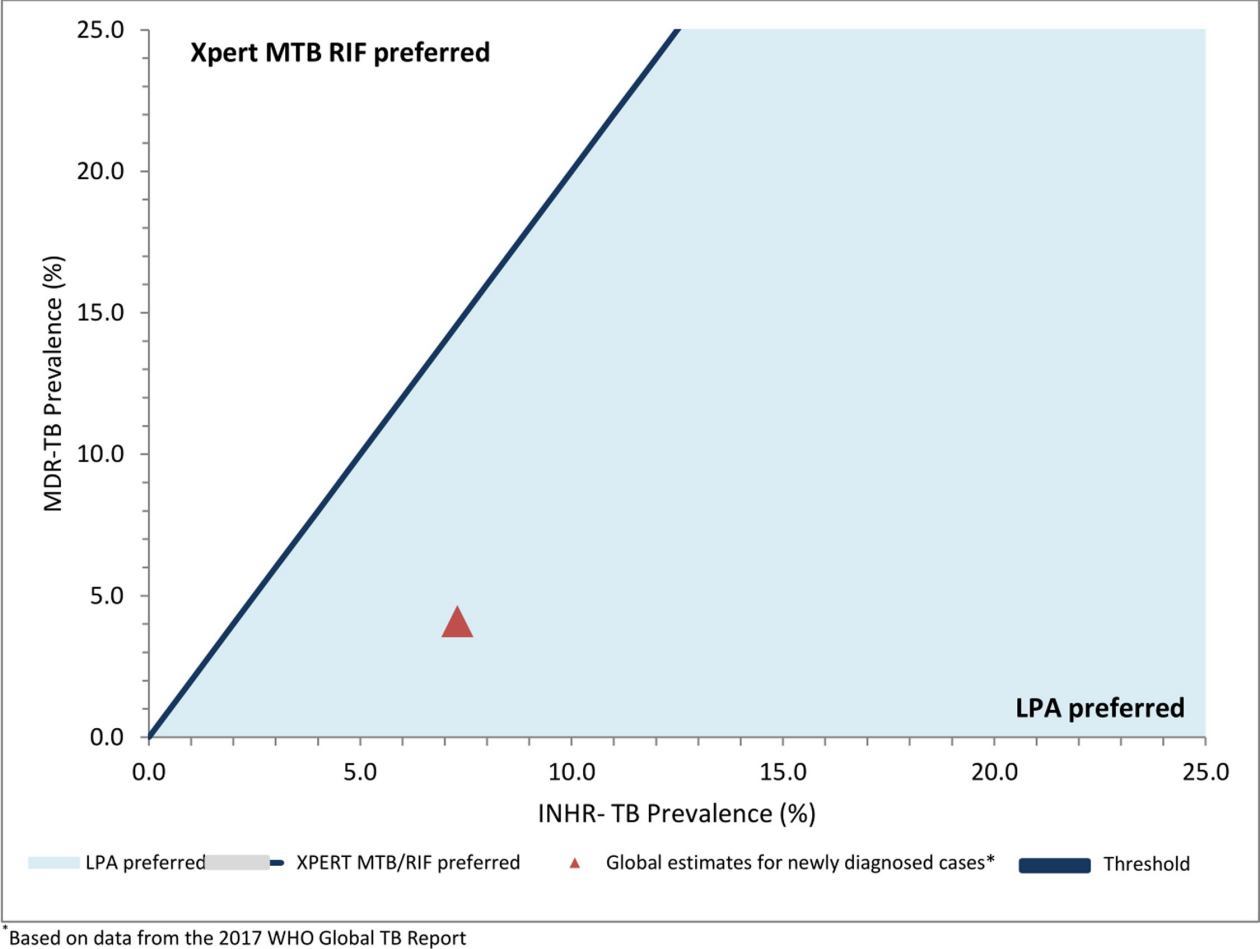
Relationship between drug resistance prevalence and diagnostic test to minimize total MDR-TB.

By ranging INHR-TB prevalence from 0% to 25%, we were able to determine that LPA was the preferred diagnostic and treatment strategy to minimize prevalent MDR-TB when INHR-TB prevalence was ≥3%; Xpert was the preferred diagnostic and treatment strategy when INHR-TB prevalence was <3% (Fig 2). At all levels of INHR-TB prevalence LPA-based diagnosis and treatment minimized the proportion of acquired MDR-TB (Fig 3). By varying both INHR-TB and MDR-TB prevalence simultaneously, it was found an Xpert-based diagnostic strategy minimized MDR-TB in settings where MDR-TB prevalence was approximately 2.0-fold higher than INHR-TB prevalence (Fig 4); in all other settings, an LPA-based diagnosis was preferred. When varying the proportion of TB patients initiating therapy based on clinical diagnosis (0.5–30.0% for Xpert and LPA-based diagnosis and treatment strategies), LPA-based diagnosis and treatment strategies resulted in lower proportions of MDR-TB and acquired MDR-TB at all levels, however, mortality in the LPA strategy remained slightly higher, indicating this assumption did not significantly impact conclusions.

### Sensitivity analysis

When comparing LPA with an Xpert-based assay that included detection of INHR-TB, the Xpert-based strategy resulted in a slightly lower proportion of death (2.92% vs. 2.89%) and prevalent MDR (1.61% vs. 1.57%). Acquired MDR was 0.35% for both detection and treatment strategies. These results are presented in S3 Table.

## Discussion

We projected epidemiologic outcomes in a globally representative cohort of patients undergoing diagnosis and treatment using three diagnostic and treatment strategies. In this model, LPA-based diagnosis combined with tailored INHR-TB treatment resulted in the lowest proportion of prevalent and acquired MDR-TB in a post-treatment population when compared with AFB smear- and Xpert MTB/RIF-based diagnostic and treatment strategies.

Notably, in all three strategies most acquired MDR-TB was generated from individuals with pre-existing INHR-TB. In both the AFB smear and Xpert MTB/RIF diagnosis and treatment strategies, approximately 80% of the acquired MDR-TB developed in patients with INHR-TB. In the LPA-based strategy, 54% of acquired MDR-TB emerged from individuals with INHR-TB. This data is consistent with recent whole genome sequencing studies demonstrating that INHR-TB usually precedes the development of MDR-TB.[22,23] When taken together, these findings have important implications for the global response to MDR-TB; improved detection and appropriate treatment of INHR-TB is necessary to prevent the generation of new MDR-TB strains. Indeed, the WHO has recently produced evidence-based guidance on INHR-TB that recommends tailored treatment regimens for INHR-TB. [24] These treatment recommendations, however, must be coupled with appropriate diagnostic infrastructure to impact patient care.

Previously, a dynamic modelling study performed by Denkinger *et al*. found that INHR-TB detection and treatment reduced MDR-TB prevalence from 3.8% to 3.6% over a 10-year period.[25] The authors concluded that INHR-TB detection and treatment was likely to have a minimal impact on MDR-TB epidemiology. The authors assumed, however, that only 1% of patients with INHR-TB treated with 2HRZE/4HR would acquire MDR-TB, compared to the 8% reported by Gegia *et al* in a recent systematic review.[5] Likewise, a dynamic modelling study by Kendall *et al*. relied on similar estimates of acquired MDR-TB when reporting that 96% of global MDR-TB resulted from transmission rather than acquisition. The authors reported that their model estimates were sensitive to the probability of acquired MDR-TB during treatment.[26] We found a similar absolute reduction in MDR-TB prevalence (0.2%) when comparing LPA and Xpert-based strategies, but noted an almost 50% relative decrease in acquired MDR-TB in the LPA-based diagnosis and treatment strategy when compared with the Xpert MTB/RIF strategy. With the increase in notification and treatment of TB globally, combined with MDR-TB programmatic scale-up, we expect the impact of acquired MDR-TB will likely increase.

Although an LPA-based strategy may show superior performance in preventing acquired MDR-TB, a TB program may be reluctant to overhaul their diagnostic infrastructure by replacing AFB smear or Xpert with LPAs. In a recent study published by Xie *et al*., an Xpert-based assay was tested that included detection of INHR-TB.[8] The investigators reported a sensitivity of 83.3% (95% CI 77.1–88.5%) and specificity of 99.2% (95% CI 95.6–100.0%) compared with DST as the reference standard, and even higher diagnostic accuracy when compared to sequencing-based results as the reference standard. We ran a sensitivity analysis based on these inputs, and while further research is required, the findings suggest that an Xpert-based platform with the ability to detect both RIFR and INHR could lead to significant reductions in acquired MDR-TB.

A number of factors limited our analysis. First, the data used represents a global distribution of drug-resistant TB and therefore may not be applicable in regions with distinct diagnostic algorithms, INHR-TB prevalence, or treatment outcomes. We performed sensitivity analysis to reflect different epidemiologic settings (Fig 4) but did not simulate the numerous regional diagnostic and treatment algorithms. This is particularly relevant in certain regions of Eastern Europe, where rates of INHR-TB are more than double the global average. [4] Results from our threshold analyses indicate that in these regions, using a strategy that incorporates accurate diagnosis and tailored treatment of INHR-TB will lead to an almost 70% relative decrease in acquired MDR-TB, when compared with an Xpert MTB/RIF diagnosis and treatment strategy.

Second, our model made several assumptions. We assumed that all patients had TB, so the impact of false positives could not be evaluated. We did, however, evaluate the implications of false negative test results. We assumed that for patients with a false negative result, a maximum of two additional opportunities for diagnosis occurred. In reality, a patient may visit a clinic several times over the course of several months before TB is diagnosed.[27] Our assumption may have enhanced the impact of superior Xpert sensitivity, and decreased the impact of an LPA-based diagnosis. To evaluate this assumption, we performed a sensitivity analysis that increased the number of patients initiating therapy based on clinical diagnosis, which reduced the impact of enhanced Xpert sensitivity; however, mortality due to undiagnosed TB in the LPA strategy remained slightly higher. Additionally, we relied on published data for parameter estimates, which were subject to substantial variability. Where possible, we used data from systematic reviews to allow for the most robust estimates of diagnostic accuracy and treatment outcomes. Finally, we excluded any additional first-line drug resistances from our analysis, as the impact of these drug resistances on treatment outcomes is less clear from the literature.

## Conclusions

In this study, using globally representative diagnostic and treatment data, we showed that treatment of undetected INHR-TB with WHO standard first-line therapy generated the majority of acquired MDR-TB in a population of pulmonary TB patients. Accurate diagnosis and tailored treatment of INHR-TB with LPA led to an almost 50% relative decrease in acquired MDR-TB when compared with the Xpert MTB/RIF strategy, suggesting that improvement in INHR-TB detection and treatment will likely help prevent further MDR-TB generation and could help reduce MDR-TB as a public health threat. Continued reliance on diagnostic and treatment protocols that ignore INHR-TB will likely result in further generation of MDR-TB globally.

## Supporting information

S1 Table Beta distribution for model inputs. (PDF)

S2 Table Probabilistic sensitivity analysis results. (PDF)

S3 Table Sensitivity analysis results. (PDF)
