# Supplementary material for: The impact of improved detection and treatment of isoniazid resistant tuberculosis on prevalence of multi-drug resistant tuberculosis: A modelling study
Source: PLoS One. 2019 Jan 24;14(1):e0211355. doi: 10.1371/journal.pone.0211355 (PMC6345486; doi:10.1371/journal.pone.0211355)
Supplement: S1 Table — (PDF) [file pone.0211355.s001.pdf]

Table S1. Beta Distribution for Model Inputs

Description	Beta Distribution
**Population proportions**	
Proportion of patients with INHR-TB	(86.14, 1078)
Proportion of patients with RIFR-TB	(21.94, 513.07)
**Diagnostic parameters**	
Sensitivity for diagnosing pulmonary TB	
AFB Smear microscopy (3 slides)	(60.65, 25.99)
Xpert MTB/RIF	(238.92, 29.53)
LPA	(24.07, 4.25)
Sensitivity for detecting rifampin resistance	
Xpert MTB RIF	(95.76, 5.04)
LPA	(1124.08, 38.36)
Specificity for detecting rifampin resistance	
Xpert MTB RIF	(10.88, 533.17)
LPA	(28.13, 2315.68)
Sensitivity for detecting isoniazid resistance	
LPA	(830.48, 90.23)
Specificity for detecting isoniazid resistance	
LPA	(21.63, 2682.51)
Clinical diagnosis, for those with a false negative diagnostic test	
AFB Smear	(21.34, 49.8)
Xpert MTB/RIF	(2.5, 47.5)
LPA	(2.5, 47.5)
**Treatment outcomes for detected or clinically diagnosed TB**	
Susceptible organism treated with standard initial treatment [2HRZE/4HR(E)]	
Death	(101, 4277)
Treatment failure	(241, 9551)
Relapse	(269, 4471)
Acquired any drug resistance due to treatment failure	(12.7, 87.3)
Acquired any drug resistance due to relapse	(1.6, 98.4)
Proportion of any acquired drug resistance that is multidrug resistant	(47, 55)
Susceptible organism treated with INHR-TB treatment [6(H)REZ]	
Death	(53, 2040)
Treatment failure	(13, 1085)
Relapse	(55, 955)
Acquired any drug resistance due treatment failure	(1.4, 98.6)
Acquired any drug resistance due to relapse	(3, 9997)
Proportion of any acquired drug resistance that is multidrug resistant	(3, 8)
INHR organism treated with standard initial treatment [2HRZE/4HR(E)]	
Death	(53, 2040)

Treatment failure	(28, 182)
Relapse	(24, 147)
Acquired multidrug resistance due to treatment failure	(8, 7)
Acquired multidrug resistance due to relapse	(1, 10)
INHR organism treated with INHR-TB treatment [6(H)REZ]	
Death	(53, 2040)
Fail	(91, 2002)
Relapse	(6, 1729)
Acquired multidrug resistance due to treatment failure	(43, 47)
Acquired multidrug resistance due to relapse	(1, 4)
MDR organism treated with standard initial treatment [2HRZE/ 4HR(E)]	
Death	(29, 175)
Treatment failure	(53, 151)
Relapse	(53, 151)
MDR organism treated with WHO standard individualized MDR-TB treatment	
Death	(479, 5502)
Treatment failure	(299, 5682)
Relapse	(10.88, 533.17)
**Outcomes for untreated TB**	
Death	(23.5, 456.5)
