# Supplementary material for: The impact of improved detection and treatment of isoniazid resistant tuberculosis on prevalence of multi-drug resistant tuberculosis: A modelling study
Source: PLoS One. 2019 Jan 24;14(1):e0211355. doi: 10.1371/journal.pone.0211355 (PMC6345486; doi:10.1371/journal.pone.0211355)
Supplement: S2 Table — (PDF) [file pone.0211355.s002.pdf]

## Probabilistic sensitivity analysis results

**Table S2a. Prevalence of MDR-TB in population after initial attempt at diagnosis and treatment, per 100 000 individuals with TB**

| | Smear (95% UR) | Xpert MTB/RIF (95% UR) | LPA (95% UR) |
|---|---|---|---|
| Total MDR-TB | 3224 (3187, 3261) | 1834 (1815, 1853) | 1628 (1607, 1649) |
| % of total population | 3.22 (3.19, 3.26) | 1.83 (1.82, 1.85) | 1.63 (1.61, 1.65) |
| Breakdown of total MDR-TB | | | |
| Ongoing undetected MDR-TB | 753 (735, 766) | 364 (358, 371) | 513 (496, 530) |
| MDR-TB cases who failed or relapsed | 1191 (1173, 1209) | 293 (289, 297) | 266 (262, 270) |
| MDR-TB cases who defaulted[a] | 733 (722, 745) | 575 (568, 583) | 548 (540, 555) |
| Acquired MDR-TB | 547 (538, 555) | 602 (592, 611) | 301 (298, 305) |

[a] Default represents a combined programmatic outcome of default, lost to follow-up or transferred out

**Table S2b. Total acquired MDR-TB in those with detected TB after initial attempt at diagnosis and treatment, per 100 000 individuals with TB**

| | Smear (95% UR) | Xpert MTB/RIF (95% UR) | LPA (95% UR) |
|---|---|---|---|
| Total TB detected or clinically diagnosed | 80785 (80574, 81105) | 90712 (90502, 90822) | 86955 (86583, 87327) |
| Total acquired MDR-TB | 547 (538, 555) | 602 (592, 611) | 301 (298, 305) |
| % of total TB detected | 0.68 (0.67, 0.69) | 0.66 (0.65, 0.67) | 0.35 (0.34, 0.35) |
| Breakdown of total acquired MDR-TB resistance due to: *(% of total acquired MDR-TB)* | | | |
| Susceptible organism treated with susceptible TB therapy | 129 (127, 132) | 143 (140, 145) | 137 (134, 139) |
| Susceptible organism treated with INHR-TB therapy | NA | NA | 0 (0,0) |
| INHR organism treated with susceptible TB therapy | 417 (409, 426) | 459 (450, 468) | 42 (41, 43) |
| INHR organism treated with INHR-TB therapy | NA | NA | 122 (120, 124) |

**Table S2c. Total death in population after initial attempt at diagnosis and treatment, per 100 000 individuals with TB**

| | Smear (95% UR) | Xpert MTB/RIF (95% UR) | LPA (95% UR) |
|---|---|---|---|
| Total death | 3206 (3188, 3225) | 2885 (2871, 2898) | 2927 (2910, 2943) |
| % of total population | 3.21 (3.19, 3.23) | 2.89 (2.87, 2.90) | 2.93 (2.91, 2.94) |
| Breakdown of total death | | | |
| Death due to undetected TB | 935 (919, 951) | 453 (445, 461) | 634 (614, 654) |
| Death during TB therapy | 2271 (2256, 2286) | 2432 (2420, 2445) | 2293 (2278, 2308) |
