# Supplementary material for: The impact of improved detection and treatment of isoniazid resistant tuberculosis on prevalence of multi-drug resistant tuberculosis: A modelling study
Source: PLoS One. 2019 Jan 24;14(1):e0211355. doi: 10.1371/journal.pone.0211355 (PMC6345486; doi:10.1371/journal.pone.0211355)
Supplement: S3 Table — (PDF) [file pone.0211355.s003.pdf]

Sensitivity Analysis: Using an Xpert-based assay that can detect both RIFR-TB and INHR-TB

Table S3a. Total MDR-TB in population after initial attempt at diagnosis and treatment, per 100 000 individuals with TB

	AFB Smear	Xpert MTB/RIF	Xpert MTB/RIF/INH	LPA
Total MDR-TB	3212	1835	1574	1614
% of total population	3.21	1.84	1.57	1.61
Breakdown of total MDR-TB				
Ongoing undetected MDR-TB	737	367	367	500
MDR-TB cases who failed or relapsed	1190	292	304	265
MDR-TB cases who defaulted[a]	733	572	574	547
Acquired MDR-TB	552	604	329	302

[a] Default represents a combined programmatic outcome of default, lost to follow-up or transferred out

Table S3b. Total acquired MDR-TB in those with detected TB after initial attempt at diagnosis and treatment, per 100 000 individuals with TB

	AFB Smear	Xpert MTB/RIF	Xpert MTB/RIF/INH	LPA
Total TB detected or clinically diagnosed	81100	90594	90594	87169
Total acquired MDR-TB	552	604	329	302
% of total TB detected	0.68	0.67	0.35	0.35
Breakdown of total acquired MDR-TB resistance due to				
Susceptible organism treated with susceptible TB therapy	129	141	140	136
Susceptible organism treated with INHR-TB therapy	NA	NA	0	0
INHR organism treated with susceptible TB therapy	423	463	71	43
INHR organism treted with INHR-TB therapy	NA	NA	118	123

Table S3c. Total death in population after initial attempt at diagnosis and treatment, per 100 000 individuals with TB

	AFB Smear	Xpert MTB/RIF	Xpert MTB/RIF/INH	LPA
Total death	3206	2889	2890	2924
% of total population	3.21	2.89	2.89	2.92
Breakdown of total death				
Death due to undetected TB	925	461	461	628
Death during TB therapy	2281	2428	2429	2296
